# Multi-Dimensional Assessment of Low-Carbon Engineering Cement-Based Composites Based on Rheological, Mechanical and Sustainability Factors

**DOI:** 10.3390/ma19020424

**Published:** 2026-01-21

**Authors:** Zhilu Jiang, Zhaowei Zhu, Deming Fang, Chuanqing Fu, Siyao Li, Yuxiang Jing

**Affiliations:** 1 College of Civil Engineering, Zhejiang University of Technology, Hangzhou 310014, China; zljiang@zjut.edu.cn (Z.J.); 18858274004@163.com (Z.Z.); chqfu@zjut.edu.cn (C.F.); lisiyao@zjut.edu.cn (S.L.); 2 College of Urban Construction, Zhejiang Shuren University, Hangzhou 310015, China; 3 Central Research Institute of Building and Construction, Co., Ltd., MCC Group, Beijing 100088, China; jingyuxiang@cribc.com; 4 Inspection and Certification, Co., Ltd., MCC Group, Beijing 100088, China

**Keywords:** LC^3^, ECC, fiber mixing, rheology, fracture distribution, carbon emissions

## Abstract

**Highlights:**

**What are the main findings?**

- Replacing OPC with LC^3^ significantly reduced carbon emissions by 19.1–20.8%.
- The cost of PP fiber used in ECC was approximately 50% of that of PVA fiber.

**What are the implications of the main findings?**

- LC^3^-ECC with PE fibers has superior tensile performance with an ultimate strain of 7.78%.
- The radar assessment can be used to optimize the mixture proportioning of low-carbon ECC.

**Abstract:**

To address the high-carbon emissions associated with the large use of Portland cement (PC) in traditional engineered cementitious composites (ECCs) and the resource constraints on supplementary cementitious materials (SCMs), this study proposes a strategy combining limestone calcined clay cement (LC^3^) as a PC replacement with the incorporation of hybrid synthetic fibers to develop low-carbon, environmentally friendly ECCs. The fundamental properties of the LC^3^-ECC were tested, and a sustainability analysis was conducted. The experimental results show that an increase in water-to-binder ratio (W/B) or superplasticizer (SP) dosage significantly enhanced fluidity while reducing the yield stress and plastic viscosity. An LC^3^-ECC with a W/B of 0.25, 0.45% SP and 2% polyethylene fibers exhibited the best tensile performance, achieving an ultimate tensile strain of 8.40%. In contrast, an increase in polypropylene fiber led to a degradation in crack-resistant properties. In terms of sustainability, replacing the PC with LC^3^ significantly reduced carbon emissions by 19.1–20.8%, while the cost of the limestone calcined clay cement–polypropylene fiber (LC^3^-PP) was approximately 50% of that of the limestone calcined clay cement–polyvinyl alcohol fiber (LC^3^-PVA). Furthermore, an integrated evaluation framework encompassing rheological, mechanical and environmental factors was established using performance radar charts. The dataset on the performance results and the developed assessment framework provide a foundation for optimizing the mixture proportioning of LC^3^-ECC in practical engineering applications.

## 1. Introduction

The cement industry produces about 8–9% of global anthropogenic carbon dioxide (CO_2_) emissions each year and is the third-largest energy consumer of human industries. In order to reduce CO_2_ emissions and energy consumption in the cement industry, it is urgently needed to develop more low-carbon cementitious materials. Previous studies mainly focus on carbon reduction efficiency and mechanical properties, but a comprehensive assessment of the performance of low-carbon materials including their rheological properties is still lacking for practical applications.

The conventional engineered cementitious composite (ECC) mix [1] contains a high content of ordinary Portland cement (OPC), resulting in high energy consumption and carbon emissions. The use of supplementary cementitious materials (SCMs) [2,3,4,5] in ECCs can greatly reduce its impact on the environment. However, the resources of SCMs such as fly ash (FA) and slag are limited due to the low production of coal-fired power, which is gradually being reduced around the world for environmental protection. Other green biomass SCMs derived from agricultural wastes, such as rice husk ash and bagasse, are not well studied and cannot be applied on a large scale due to decentralized collection and economic unviability. In this context, kaolinite clay and limestone powder with abundant proven reserves globally are emerging as a potential substitute for Portland cement.

Limestone calcined clay cement (LC^3^) is a low-clinker cement, and its main components are Portland cement clinker, calcined clay, limestone and gypsum. The calcination temperature of clay (600–800 °C) is significantly lower than the calcination demand of Portland cement clinker. Moreover, the resulting metakaolin exhibits superior pozzolanic activity, enabling substantially higher clinker substitution rates. Sánchez Berriel et al.’s [6] results indicate that LC^3^ achieves 22% lower energy consumption and 20–30% reduced CO_2_ emissions during production versus conventional Portland cement, without compromising concrete workability, strength or durability. LC^3^-based ECCs are recognized as a highly promising cementitious material due to their low-carbon footprint, environmentally sustainability, enhanced ductility and comparable strength [7,8,9]. Zhang et al. [10] found that replacing OPC with LC^3^ in ECCs enhances its early-age strength. However, due to the high water demand of calcined clay during mixing, the water-to-binder ratio is increased by 20%, which leads to a lower 28-day compressive strength [11,12]. The tensile strain capacity of LC^3^-based ECCs exceeds 6%, and the average residual crack width is below 50 μm. LC^3^-based ECCs improved fiber–matrix interfacial bond strength and pull-out energy. Liang et al. [13] studied the macroperformance and micromechanism of LC^3^- ECCs and found that LC^3^ substitution led to more hydration products and higher fiber/matrix bonding, which improved multiple cracking behavior. Qi et al. [14] studied the effects of FA proportion on the hydration, microstructure and mechanical properties of LC^3^-based ECCs. The addition of FA reduced the calcium hydroxide content and limited overall hydration, resulting in increased total porosity and reduced strength. While the increase in FA proportion decreased the bonding strength between the fiber and matrix, the ductility and fracture toughness of the LC^3^-based ECC were significantly improved. High volumes of FA, typically with FA/LC^3^ ratios of 1.0 to 1.5 [15,16], were used in LC^3^- ECC systems due to its improvement in strain-hardening capacity and reduction in carbon emissions.

As the primary reinforcement in ECCs, fibers enable crack-width control via bridging effects, which improves cracking behavior and tensile ductility [17]. Ma et al. [18] confirm that incorporating 0.25 vol% polyvinyl alcohol (PVA) fibers increases the splitting tensile strength of cementitious matrices by 25%. The low elastic modulus of PVA fibers facilitates tensile strengths exceeding 5 MPa and ultimate strains exceeding 3%. The high cost of PVA fibers impedes their large-scale engineering application in ECCs, shifting research focus toward hybrid fiber reinforcement systems. Hybrid fibers may have advantages that a single-fiber system does not have at a specific proportion [15]. For instance, hybridizing polypropylene (PP) and PVA fibers maintains the ECC’s ductility while reducing material costs. Lin et al. [19] studied the mechanical behavior of hybrid PP-PVA ECCs. Increasing PP fiber content reduced compressive strength while enhancing tensile strength and strain capacity. But the tensile strength and impact resistance remained marginally inferior to the PVA-ECC.

In addition to the assessment of the basic properties of the materials, the multi-dimensional evaluation of ECCs also includes the use of a life cycle assessment (LCA) for environmental decision making. Life cycle analysis can be used to evaluate the environmental impact of cement-based materials through the material sustainability indicator (MSI), which mainly includes the embodied energy and carbon footprint. Choi et al. [20] found that a mixture with selvage-based short fibers showed higher sustainability compared to that of a mixture with normal PE fibers based on material sustainability indicators. In the development of an OPC-PP-ECC, Zhu et al. [21] used PP fiber instead of PVA to reduce the implicit energy by 150%. By replacing OPC with LC^3^, the carbon footprint was reduced to 52% of the OPC-ECC. In order to evaluate the overall performance of high-strength ECCs with a high content of ternary cementitious materials (fly ash–limestone–calcined clay), Younas et al. [22] compared seven key performance indicators including mechanical properties, environmental impact and economy. Hou and Li [15] found that the embodied energy and carbon footprint of PVA fibers were 31% and 10% higher than those of PP fibers, respectively, and the cost was doubled. Compared with the single PVA-ECC, the total cost, embodied energy and carbon footprint of the PVA/PP-ECC were reduced by 25.5%, 10.7% and 3.2%, respectively.

However, there is a lack of overall research on the multi-dimensional assessment of the rheology, mechanical properties and environmental impact of different synthetic fibers (PVA/PP and PE/PP) in the LC^3^ matrix. Based on this, this study explores the influence of water-to-binder ratio, superplasticizer (SP) dosage and PP fiber hybridization rate on the fluidity and mechanical properties of ECCs for low-carbon synthetic fiber composite systems and uses an LC^3^-ECC performance radar chart to form a set of ECC performance evaluation systems. Compared with previous studies, the comprehensive material and sustainability performance of the LC^3^-based ECC were considered via a multi-dimensional assessment, which can be conveniently used for decision making regarding composite mixes in future applications.

## 2. Experimental

### 2.1. Materials and Specimen Preparation

**Figure 1:**
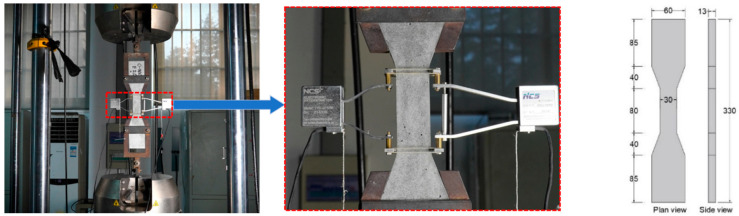
ECC tensile specimen dimensions and tensile test.

Limestone calcined clay cement (LC^3^) cooperating with fly ash (FA) was used as a green binder for the ECC. The raw materials for the cementitious matrix include PI 42.5 Portland cement (PC), FA, metakaolin (MK), limestone powder (LS) and quartz sand (QS) with 70 to 110 mesh. Their chemical composition is shown in Table 1. Polyvinyl alcohol (PVA) fiber, ZTD25 polyethylene (PE) fiber and polypropylene (PP) fiber were used as reinforcement. Among them, the PP fiber incorporated 0.2% antioxidant and 8% oil treatment during production. The geometric and physical parameters of the fibers are shown in Table 2. Polycarboxylate superplasticizer (SP) DFTR-PCE and deionized water was used. The mix proportion of the ECC mortar matrix is presented in Table 3 with different water-to-binder ratios and superplasticizer dosage. PVA, PE and hybrid PVA/PP and PE/PP fibers were used, and the fiber volume fraction was 2%. For group numbers, “O” represents an OPC matrix, “L” represents an LC^3^ matrix, “W” represents the water-to-binder ratio, “S” represents the superplasticizer dosage, “A” represents PVA fiber, “E” represents PE fiber and “P” represents PP fiber. For example, L-W25S40-E1.5P denotes an LC^3^ matrix, a water-to-binder ratio of 0.250, a superplasticizer (SP) dosage of 0.40% and a PE content of 1.5% (PP substitution 0.5%). Prism specimens (40 mm × 40 mm × 160 mm) were cast for flexural and compressive strength testing; dog-bone-shaped specimens with dimensions shown in Figure 1 were employed for direct tensile tests. All specimens were demolded 24 h after casting and cured for 28 days at 20 ± 3 °C and >95% relative humidity.

### 2.2. Flowability and Rheological Tests

The flowability of fresh ECC paste was determined according to the Chinese standard GBT-2419-2005 [23]. The freshly mixed paste was poured into a cone mold in two layers, and the mold was then vertically lifted. The flow table was activated to complete 25 strokes at a frequency of 1 Hz. After that, the spread diameter was measured in two perpendicular directions, and the average value was recorded as the spread diameter.

The rheological properties of fresh mortar were measured using a Brookfield R/S-SST rheometer (Brookfield Engineering Labs, Middleborough, MA, USA). Mortar deformation was induced by the relative motion between the fixtures. The stress/strain dual-mode control system was configured to meet the test requirements of different experimental conditions. RHEO V2.8 software was used to control the rheometer, as well as for data acquisition and analysis. To intuitively compare the changes in the rheological parameters of the slurry, the Bingham model (Equation (1)) was used to fit the curve of shear rate–shear stress, and the intercept (representing yield stress) and slope (representing plastic viscosity) were obtained.

(1) $$\tau =\tau_{0}+\eta \dot{\gamma }$$

where *τ* is shear stress (Pa), *τ*_0_ is yield stress (Pa), *η* is plastic viscosity (Pa·s) and *γ* is shear rate (1/s).

### 2.3. Mechanical Property Tests

The flexural strength tests were conducted according to the Chinese standard GBT 17671-2021 [24]. The flexural strength of specimens were tested by a universal testing machine (Lushida WE-300B, Beijing Lushida Instrument Equipment Co., Ltd., Beijing, China) with a loading rate of 0.5 kN/s. The flexural strength of the specimen group was calculated as the average value of three specimens. Values deviating by more than 10% from the average were discarded, and the mean value of the remaining specimens was reported as the final flexural strength. After the flexural test, each specimen was fractured into two halves. Compressive tests were subsequently performed on each half-prism using the universal testing machine with a loading rate of 2.4 kN/s. The compressive strength of the group was determined as the average of all valid specimens, excluding those with values exceeding ±10% of the mean.

In the direct tensile test, two custom fixtures were used, and the displacement rate was 0.15 mm/min. When the tensile force of the specimen decreased to 80% of the maximum tensile force, the loading was stopped. The tensile strain in the gauge area during the loading process was measured using a clip extensometer (Beijing Zhongxi Huada Technology Co., Ltd., Beijing, China). The gauge length was 80 mm. After the uniaxial tensile test, the residual crack images of each specimen after reaching the ultimate strain were recorded. The crack width and crack density of specimens with different mix proportions were quantitatively measured using a 1600× digital electron microscope (Tiankui Electronics (Shenzhen) Group Co., Ltd., Shenzhen, China).

### 2.4. Scanning Electron Microscope Test

A Tescan Mira4 high-performance scanning electron microscope (SEM) (Tescan China, Ltd., Shanghai, China) was used to observe the fibers on a cross section of the specimen after the uniaxial tensile test. Before the SEM test, samples were prepared according to the following steps: first, the specimen was cut along the vicinity of the section, and the morphology of the section was retained as much as possible; then, the sample was placed in a vacuum drying oven for 40 °C vacuum drying to a constant weight; finally, the dried sample was sprayed with gold by a high-resolution sputtering coating instrument ETD-800 (Beijing Elaborate Technology Development Ltd., Beijing, China).

## 3. Results and Discussion

### 3.1. Fresh Properties

**Figure 2:**
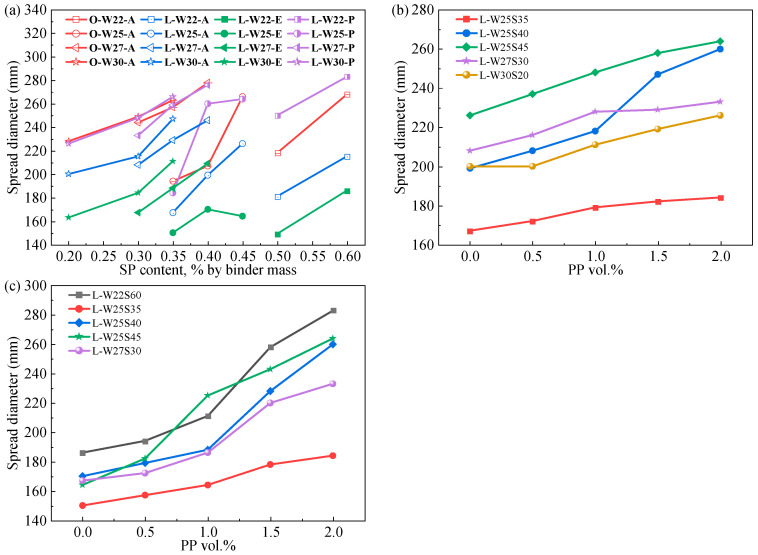
Measured fluidity: (**a**) ECC with different SP content; (**b**) PVA/PP-LC^3^-ECC with different PP content; (**c**) PE/PP-LC^3^-ECC with different PP content.

The effects of water-to-binder ratio (W/B), superplasticizer (SP) dosage and fiber type on the flowability of the ECC are shown in Figure 2. At the same SP dosage, the flowability of the ECC pastes increased with increasing W/B. At the same W/B and SP dosage, the flowability of LC^3^-ECC pastes incorporating different fiber types followed the order: PP > PVA > PE. The surface treatment of PVA fibers enhanced the paste flowability more significantly than that of hydrophobic PE fibers. As shown in Figure 2b,c, the flowability of the LC^3^-ECC increased with increasing PP fiber dosage. This is because the hydrophobic nature and surface oil treatment of PP fiber further reduce the interfacial friction with the matrix paste. The increase in the flowability of the PE/PP-LC^3^ paste due to the inclusion of PP fiber was greater than that of the PVA/PP-LC^3^ paste. The flowability of PVA-OPC is better than that of PVA-LC^3^, which is due to the high specific surface area and layered particle structure of calcined clay in LC^3^, resulting in high water demand and the promotion of greater flocculation in the paste [25].

**Figure 3:**
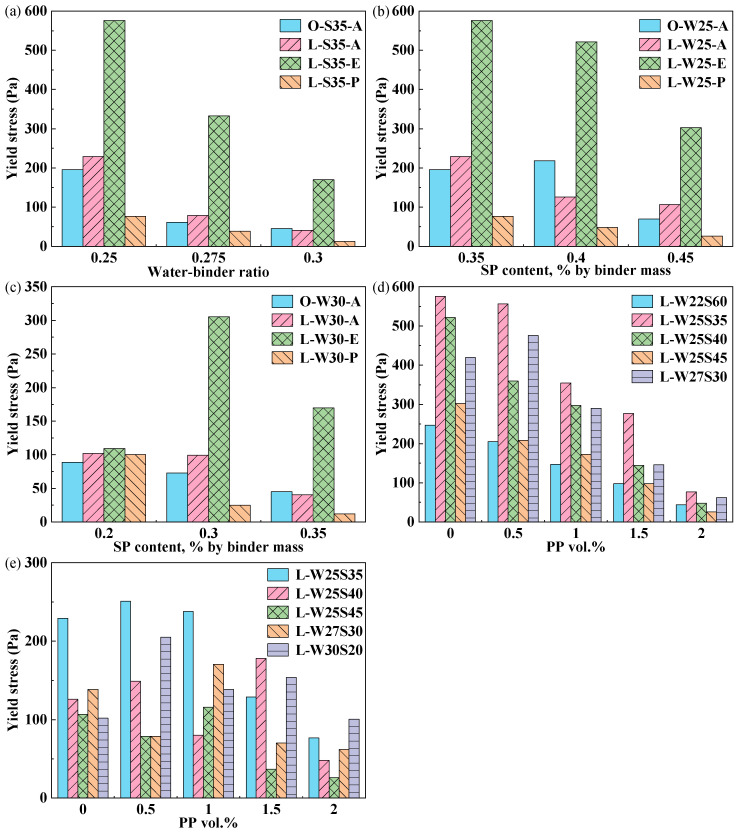
Yield stress: (**a**) SP dosage 0.35%, different W/B; (**b**) Different SP dosage, W/B 0.250; (**c**) Different SP dosage, W/B 0.300; (**d**) Different PP content in PE/PP-LC^3^; (**e**) Different PP content in PVA/PP-LC^3^.

The yield stress represents the minimum shear stress required to initiate continuous plastic flow in the paste, while the plastic viscosity characterizes the viscous resistance of the flowing paste to changes in shear rate. The measured result was fitted using the Bingham model to determine the yield stress and plastic viscosity. To analyze the effect of W/B on the shear stress of ECC, the SP dosage was fixed at 0.35%. Figure 3a shows that the yield stress decreased with an increasing W/B, which contrasted with the increase in flowability. This indicates a negative correlation between yield stress and flowability. In Figure 3a–c, when the SP dosage was smaller than 0.35%, the yield stress of the L-A group was generally higher than that of the O-A group, due to the high water demand of the calcined clay [25]. In the LC^3^-ECC, the yield stress of different fiber types is significantly different, and the yield stress decreased in the order of PE > PVA > PP. The yield stress and plastic viscosity of paste containing surface-treated PVA fibers decreased, which was less than that of PE fibers. PP fibers were also surface-treated, and the lubricating oil film formed on the PP fiber surface reduced the interfacial friction between the fibers and matrix particles, reducing the shear resistance and viscous resistance during paste flow.

**Figure 4:**
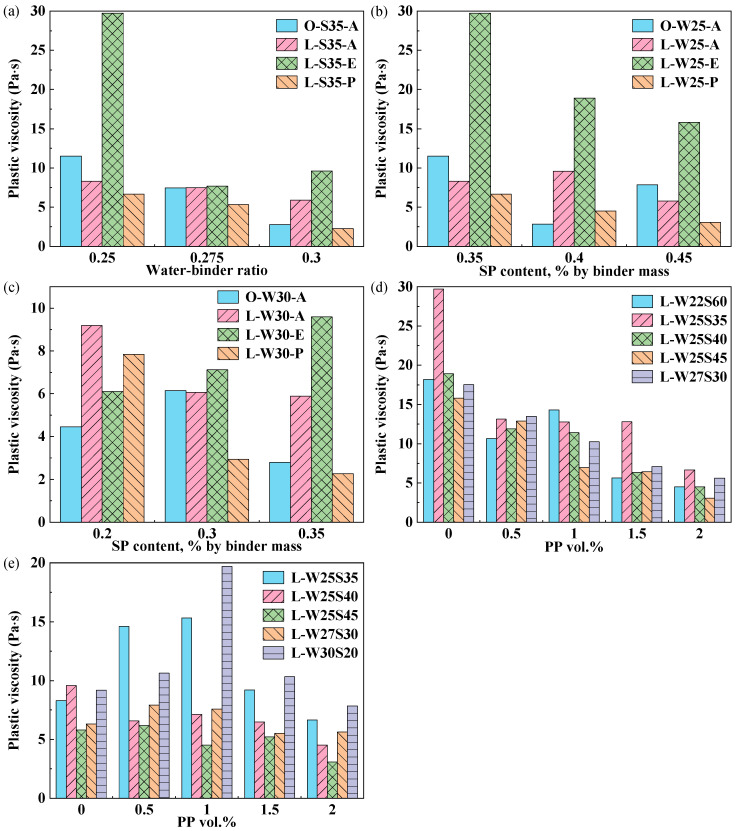
Plastic viscosity: (**a**) SP dosage 0.35%, different W/B; (**b**) Different SP dosage, W/B 0.250; (**c**) Different SP dosage, W/B 0.300; (**d**) Different PP content in PE/PP-LC^3^; (**e**) Different PP content in PVA/PP-LC^3^.

Figure 4a shows that the plastic viscosity decreased with increasing W/B, except for the L-E group at a W/B of 0.3. This reduction is attributed to the lubrication effect of free-water films, increased particle spacing and the weakening of flocculation structure with an increase in W/B [26]. As shown in Figure 3b and Figure 4b, at a W/B of 0.250, the yield stress and plastic viscosity of all ECC pastes generally show a decreasing trend as the SP dosage increases. This reduction is attributed to the dispersion of cement particles by the SP, which reduces interparticle attraction and lowers the shear resistance of the paste and flow resistance, consequently decreasing both yield stress and plastic viscosity [27]. According to the results shown in Figure 3c and Figure 4c, at a W/B of 0.300, the yield stress and plastic viscosity of the L-E group increases when the SP dosage increases from 0.20% to 0.35% due to severe paste segregation. Therefore, at a W/B of 0.300, SP dosages exceeding 0.20% are not recommended for the L-E group to avoid these adverse effects.

The yield stress and plastic viscosity of the PE/PP-LC^3^ group decreased with increasing PP fiber content, as shown in Figure 3d and Figure 4d. As shown in Figure 3e and Figure 4e, the change in the yield stress and plastic viscosity of the PVA/PP-LC^3^ group with increasing PP fiber content exhibited a more complex rheological behavior than that of the PE group. This complexity may arise from the surface treatment applied to both PVA and PP fibers, which reduces water and SP adsorption on fiber surfaces, the thickening of lubricating water films around cementitious particles, the increased concentration of free SP molecules in the aqueous phase and the heightened susceptibility to instability phenomena such as bleeding and segregation. Generally, the decrease in the yield stress and plastic strength of the PVA/PP-LC^3^ group was more significant than that of the PE/PP-LC^3^ group.

### 3.2. Compressive Strength and Flexural Strength

**Figure 5:**
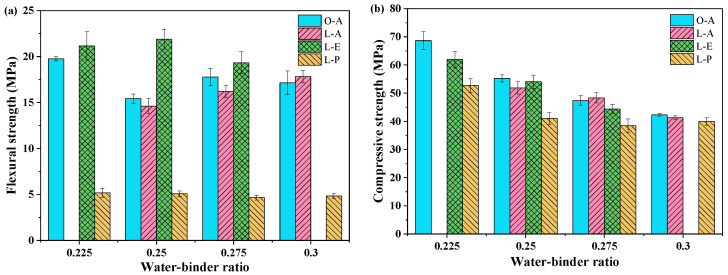
Flexural strength and compressive strength with different W/Bs: (**a**) Flexural strength; (**b**) Compressive strength.

As shown in Figure 5, compressive and flexural strength followed the order: PE-LC^3^ > PVA-OPC > PVA-LC^3^ > PP-LC^3^. There is a lack of results for the L-A mix at a W/B of 0.225 and the L-E mix at a W/B of 0.30 due to unsatisfied workability. Compressive strength decreased progressively with increasing W/Bs. At the same W/Bs and SP dosages, the flexural strength trend across fiber types corresponded fundamentally with the ranking of fiber tensile strength. The compressive and flexural strength of the PVA-LC^3^ groups were comparable to or even slightly higher than that of PVA-OPC.

**Figure 6:**
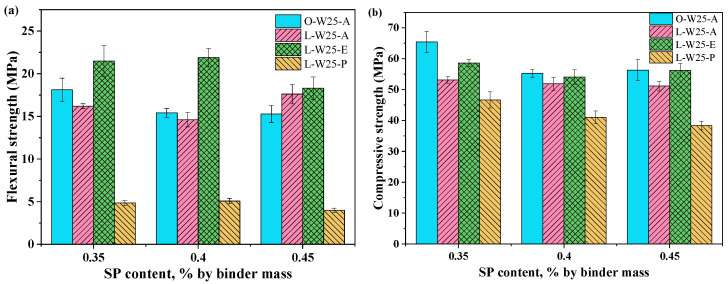
Flexural strength and compressive strength with SP dosage (W/B = 0.250): (**a**) Flexural strength; (**b**) Compressive strength.

As shown in Figure 6a, the flexural strength of the L-E group and L-P group exhibited an initial increase followed by a decrease as SP dosage increased. Figure 6b indicates that the O-A group has a decrease in compressive strength with increasing SP dosage while the L-A group shows slight changes. By improving workability, the SP promotes more uniform fiber dispersion and strengthens the fiber–matrix interfacial bond, which enables more efficient stress transfer and consequently increases flexural strength. Conversely, an excessive SP dosage may induce a lubricating layer at the fiber–matrix interface, which weakens the fiber bridging effect and can trigger gravitational sedimentation-induced inhomogeneous fiber distribution [28]. In contrast, PVA fiber-reinforced OPC composites demonstrated greater sensitivity to SP dosage and were more susceptible to excessive interfacial lubrication upon SP addition. Particularly, when the SP dosage increased from 0.35% to 0.45%, a 15.47% reduction in flexural strength was observed in the O-A group.

**Figure 7:**
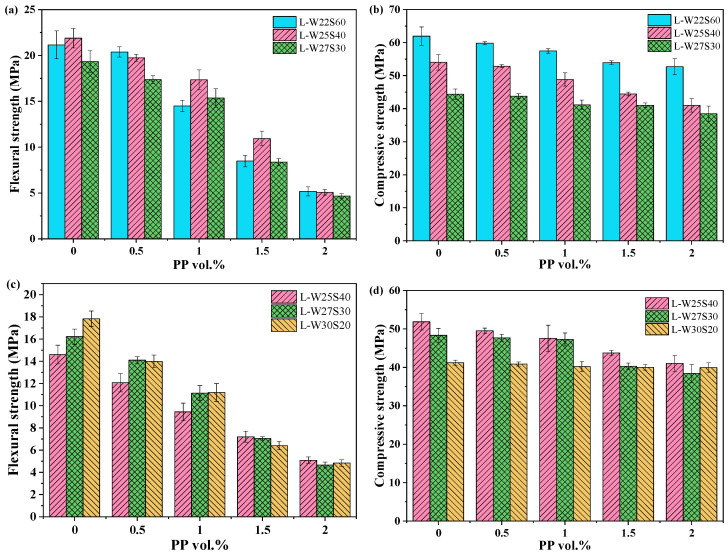
Flexural strength and compressive strength with PP content: (**a**) Flexural strength of the PE/PP-LC^3^ group; (**b**) Compressive strength of the PE/PP-LC^3^ group; (**c**) Flexural strength of the PVA/PP-LC^3^ group; (**d**) Compressive strength of the PVA/PP-LC^3^ group.

Figure 7 demonstrates that both the flexural strength and compressive strength of the PE/PP-LC^3^ group and PVA/PP-LC^3^ group decreased with increasing PP fiber content. When the PP fiber replacement increased from 0% to 100%, for the W22S60, W25S40 and W27S30 groups, the flexural strength of the LC^3^-ECC decreased by 75.53%, 76.82% and 75.89%, respectively, and the compressive strength decreased by 14.91%, 24.09% and 13.33%, respectively. For the W25S40, W27S30 and W30S20 groups, the flexural strength of LC^3^-PVA specimens decreased by 65.27%, 71.27% and 72.88%, respectively, and the compressive strength decreased by 20.88%, 20.45% and 3.19%, respectively. The results indicate that the reduction in flexural strength due to PP fiber replacement is much greater than that of compressive strength. At the same W/B and SP dosage, the LC^3^-PE composites consistently exhibited superior flexural and compressive strength performance compared to the PVA/PP-LC^3^ group.

### 3.3. Tensile Performance

**Figure 8:**
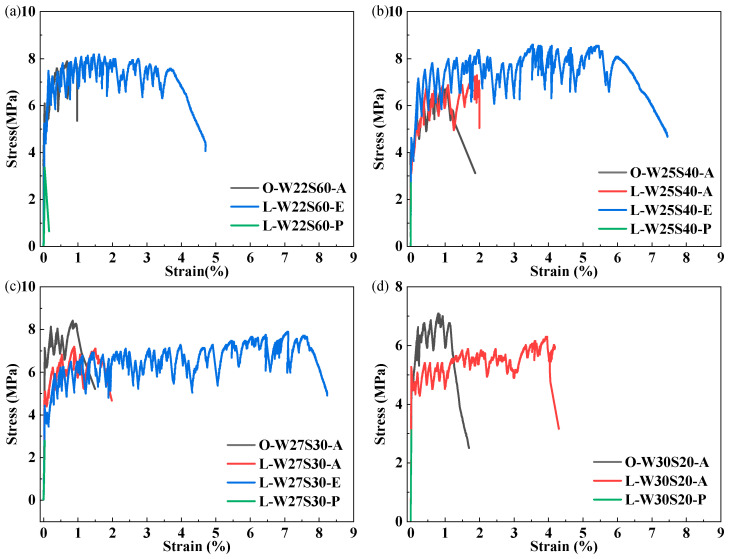
Stress–strain curves under different W/Bs: (**a**) W22S60 group; (**b**) W25S40 group; (**c**) W27S30 group; (**d**) W30S20 group.

The stress–strain curves of ECC specimens with different W/Bs under uniaxial tension are presented in Figure 8, with the measured parameters summarized in Table 4. The results demonstrate that fiber type significantly influences the tensile behavior of the LC^3^-ECC. The OPC-PVA group exhibited elevated initial crack strength due to its higher matrix strength but displayed a shortened strain-hardening stage. However, despite the lower matrix strength of the L-E group, it exhibited extended strain-hardening stages and greater ultimate tensile strains. The L-W27S30-E group achieved an ultimate tensile strain of 7.78%. These findings demonstrate a positive correlation between matrix strength and cracking strength, while revealing an inverse correlation between matrix strength and ductility [17].

**Figure 9:**
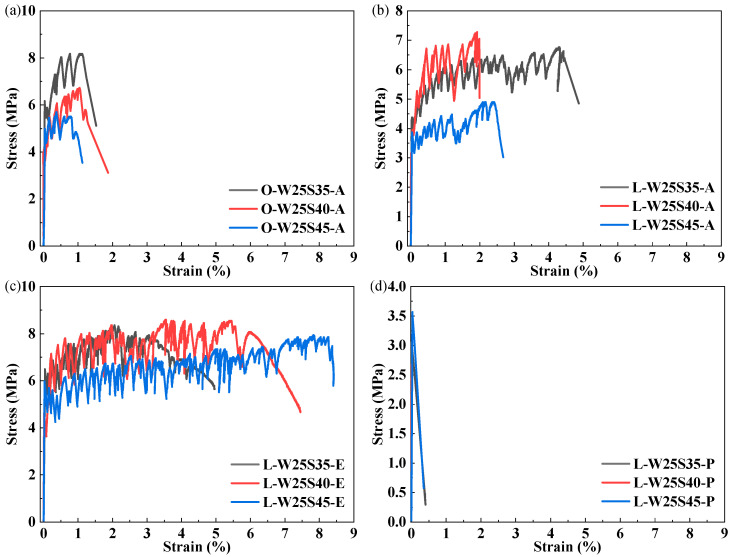
Stress–strain curves by dosage of SP: (**a**) O-A group; (**b**) L-A group; (**c**) L-E group; (**d**) L-P group.

As shown in Figure 9, at a W/B of 0.25, increasing the SP dosage from 0.35% to 0.45% induced different responses in the tensile properties of ECCs, and the corresponding tensile performance parameters are summarized in Table 5. The initial cracking strength, ultimate strength and ultimate tensile strain of the OPC-PVA group decreased by 19.0%, 30.5% and 15.9%, respectively, as SP dosage increased to 0.45%.

**Figure 10:**
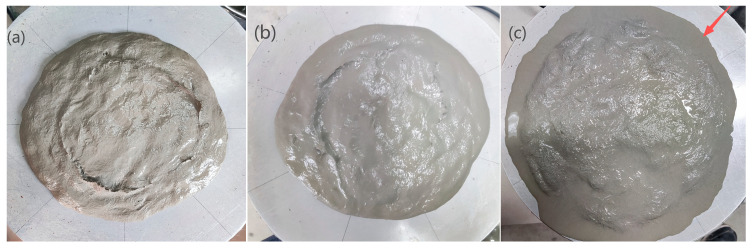
Fluidity tests of the OPC-PVA group with different SP dosages: (**a**) O-W25S35-A; (**b**) O-W25S40-A; (**c**) O-W25S45-A.

The performance degradation of the OPC-PVA group was related to bleeding and segregation, indicated by abnormal rheological parameters due to SP overdose. Figure 10 illustrates that obvious bleeding at the outer layer of the paste was observed when the SP dosage increased from 0.35% to 0.45%. The LC^3^-PVA composite achieved a peak ultimate tensile strain (4.47%) at the 0.35% SP dosage. Excessive SP (>0.35%) reduced the strain to 2.00–2.42%, attributed to increased lubricating water film thickness and enhanced steric hindrance effects. The LC^3^-PE group exhibited an ultimate tensile strain of 8.40% at an SP dosage of 0.45%, but the strength decreased slightly. The LC^3^-PP group consistently demonstrated brittle fracture behavior, indicating insufficient fiber–matrix interfacial bonding.

**Figure 11:**
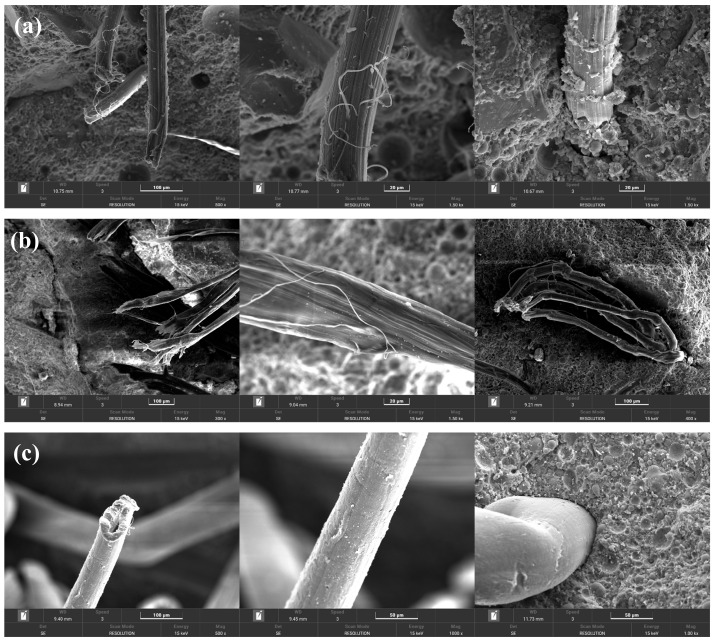
Surface morphologies of the LC^3^-ECC group with different types of fibers: (**a**) L-W25S40-A; (**b**) L-W25S40-E; (**c**) L-W25S40-P.

Figure 11 shows the post-fracture surface micromorphology of ECC specimens with different fiber types. Figure 11a,b indicate that compared to PVA fibers, PE fibers exhibit less surface damage, with fewer scratches and less fraying on the fiber surface. Due to the hydrophobicity of PE fibers, the chemical bond energy between the fibers and matrix is weaker than that of PVA fibers. The moderate bonding strength promotes controlled slippage of the PE fibers within the matrix, which not only effectively transfers stress but also dissipates energy through slippage. However, when examining fiber distribution, an occasional non-uniform dispersion of PE fibers was observed. As shown in Figure 11c, the surface of PP fibers is relatively smooth with minimal scratches. Similar to PE fibers, PP fibers have fewer hydration products attached. Gaps exist at the interface between PP fibers and the matrix, further confirming the lower bonding strength. Additionally, radial cracks extending outward are observed at the roots of the PP fibers. PP fibers undergo oil treatment to facilitate dispersion, and this treatment, combined with their hydrophobicity, appears to synergistically reduce the bonding between PP fibers and the matrix. The lower bonding strength causes specimens with PP fibers to have pull-out failure rather than fracture after axial tensile testing.

**Figure 12:**
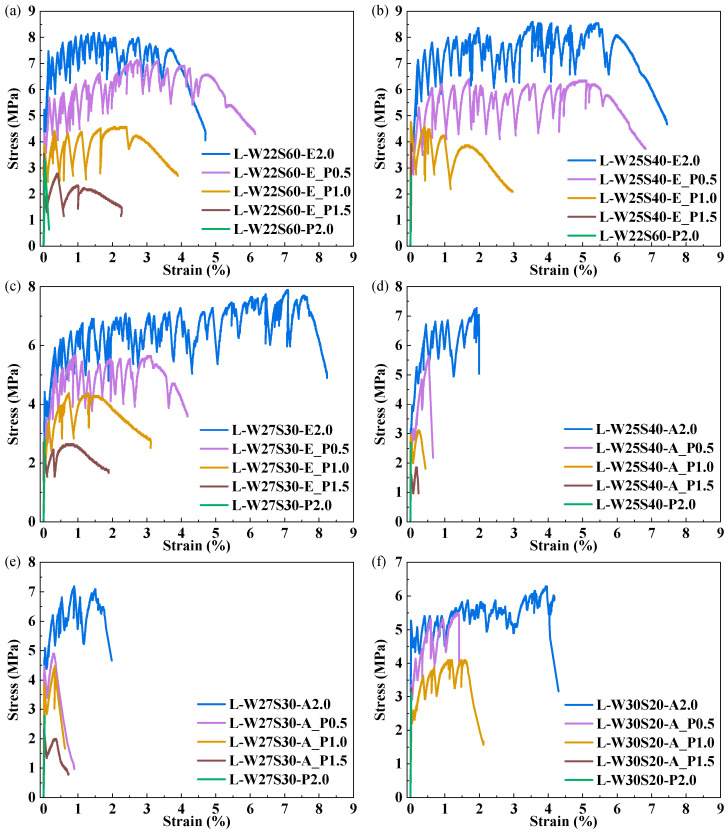
Stress–strain curves with a change in PP content. (**a**) PE-W22S60; (**b**) PE-W25S40; (**c**) PE-W27S30; (**d**) PVA-W25S40; (**e**) PVA-W27S30; (**f**) PVA-W30S20.

The uniaxial tensile stress–strain curves for LC^3^-PE and LC^3^-PVA composites with different PP fiber replacement are presented in Figure 12, with the measured tensile properties presented in Table 6. In Figure 12, a progressive reduction in tensile strength was observed in both LC^3^-PE and LC^3^-PVA systems with increasing PP fiber content. The reduction in tensile strength was probably due to inferior fiber/matrix interfacial properties according to Ahmed et al.’s work [29]. Exceptionally, the comparison between L-W22S60-E and L-W22S60E1.5P shows that a 48.11% increase in ultimate tensile strain was achieved at 0.5% PP fiber replacement. This enhancement is attributed to the improved paste rheology of the low W/B (0.225) matrix when PP fiber was included. However, at higher PP fiber replacement (≥1.0%), tensile performance deterioration was observed due to the inherently lower tensile strength of PP fibers. The ultimate tensile strength of the LC^3^-PVA composite was marginally lower than that of the LC^3^-PE system, and the ultimate elongation of the LC^3^-PVA group is basically less than 2.0%. However, at a W/B of 0.300, reduced matrix strength in the LC^3^-PVA group promoted multiple crack propagation, resulting in an ultimate tensile strain of 4.20%. Compared with PE fiber, PVA fiber can contribute to resisting cracking at lower load levels.

### 3.4. Residual Crack-Width Distribution

After concrete cracks, water and chloride ions can transport into the material much faster, which impacts the durability. Crack width is positively correlated with the transport rates of the corrosive species. The probability density function (PDF) and cumulative distribution function (CDF) for crack width were represented as the Weibull distribution given by Equations (2) and (3), respectively:

(2) $$f_{w}(w)=\left(\frac{k}{\lambda }\right){\left(\frac{w}{\lambda }\right)}^{k-1}\exp\left(-{\left(\frac{w}{\lambda }\right)}^{k}\right)$$

(3) $$F_{W}\left(w\right)=1-\exp\left(-{\left(\frac{w}{\lambda }\right)}^{k}\right)$$

where *f_w_*(*w*) is the probability density function of the Weibull distribution; *F_w_*(*w*) is the cumulative distribution function of the Weibull distribution; *w* is crack width; *λ* is the scale parameter and *k* is the shape parameter. As the parameter *λ* increases, the overall distribution profile shifts to the right, indicating a larger mean width. The tails of the distribution become heavier with a larger parameter *k*, indicating a greater probability of extreme values.

**Figure 13:**
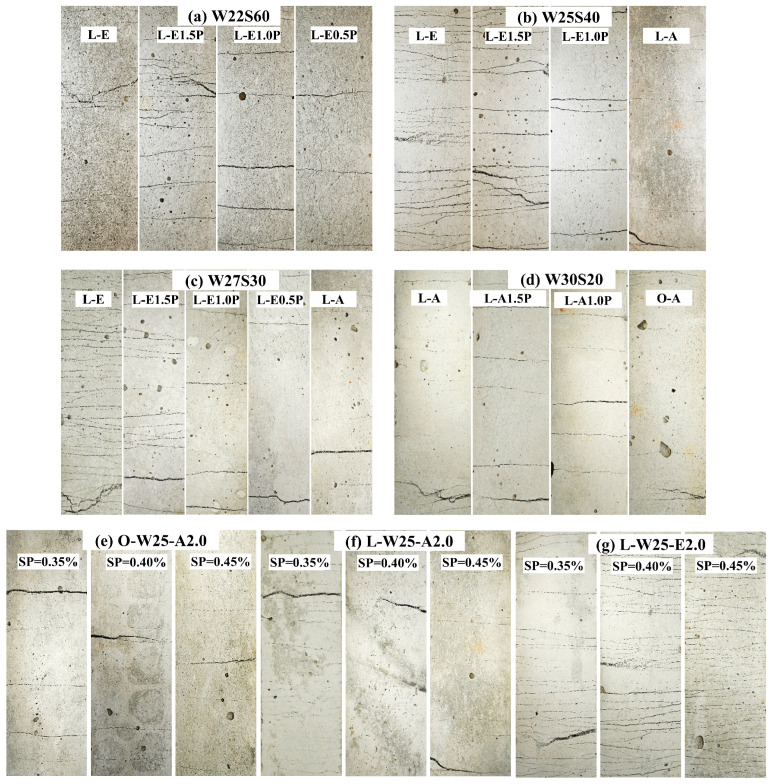
Images of cracks after tensile tests: (**a**) W22S60; (**b**) W25S40; (**c**) W27S30; (**d**) W30S20; (**e**) O-A(W25); (**f**) L-A(W25); (**g**) L-E(W25).

Figure 13 shows the crack diagrams after the tensile tests. Figure 13a–d show the change in the crack distribution of LC^3^-PE/PVA specimens with an increase in PP replacement. With an increase in PP content, the number of cracks in the LC^3^-PE decreased significantly, and the average crack spacing increased. When PE fiber was completely replaced by PP fiber, the LC^3^-ECC specimen lost ductility with brittle fractures. Compared to the water–binder ratios of 0.25 and 0.275, the number of cracks in LC^3^-PVA specimens increased significantly when the water–binder ratio was 0.30. When the water–binder ratio was 0.30, multi-crack cracking patterns were still preserved with the substitution of PP fibers, as shown in Figure 13d. In addition, the cracking mode of the LC^3^-PVA is better than that of the OPC-PVA with a greater number of microcracks.

The residual crack-width distribution of different specimen groups is presented in Table 7, and the confidence interval for the parameter with a 95% confidence level is shown in Table 8. The fitted distribution parameters and corresponding correlation coefficients (R) are also presented. The R-values for all specimens ranged from 0.826 to 0.988, demonstrating the applicability of the Weibull distribution for characterizing crack-width distributions in LC^3^/OPC-ECC systems across varying fiber hybridization ratios. For the W22S60 group, the parameters λ and *k* both increase with increased PP fiber replacement, indicating a larger mean cracking width and the greater probability of extreme widths. Except for the W30S20 group, the parameters λ and *k* both decrease when the OPC matrix is replaced by an LC^3^ matrix. This can be explained by a greater bonding between the fiber and LC^3^ matrix [13], which leads to improved multi-cracking behavior.

**Figure 14:**
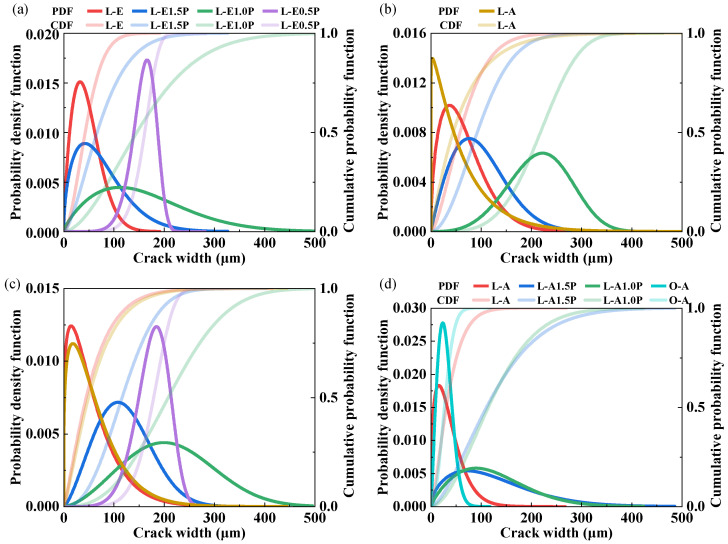
Effect of PP content on the crack-width distribution of the LC^3^-ECC: (**a**) W22S60; (**b**) W25S40; (**c**) W27S30; (**d**) W30S20.

Figure 14 shows the effect of PP content on the crack-width distribution of the LC^3^-ECC under varying W/Bs. In general, the crack-width distribution curves of PE/PVA-LC^3^ specimens move rightward, and the peak intensity declines with an increase in PP content, which is in line with the increase in average crack width, indicating that an increase in PP content would gradually reduce the fiber-mediated crack-width control. For LC^3^-PE composites with low PP fiber content (0.5%), the residual average crack width remained within the controllable range of 75.7–98.6 μm when the W/B is smaller than 0.275. Exceeding a W/B of 0.275 resulted in average crack widths surpassing 100 μm. Similarly, Hou and Li’s results [15] show that PP replacement increased average crack width, reaching 68 μm under 1% tensile strain and 105 μm under 8% tensile strain. They also indicated that when the PP replacement was 50%, the average crack width was around 50 μm under 2% tensile strain, which is the working conditions of most infrastructures. In this study, the L-W22S60-E1.5P group has a 25% PP replacement, and its average crack width reaches 75.7 μm under a tensile strain of 5.48%, which is expected to be satisfactory for infrastructure use. However, when the PP replacement increased to 50%, the average crack width greatly increased: 158.1 μm under a tensile strain of 2.76%.

**Figure 15:**
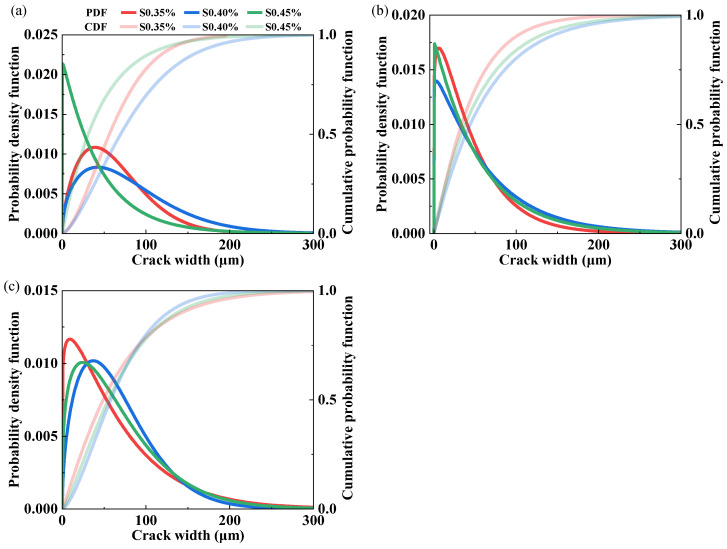
Effect of SP dosage on the crack-width distribution of ECCs: (**a**) O-A; (**b**) L-A; (**c**) L-E.

Figure 15 illustrates the influence of SP dosage on the crack-width distribution of ECCs at a W/B of 0.250. Compared to the effects of PP fiber content, SP dosage exerted a relatively minor influence on crack-width distribution. In the O-A group, a distinct leftward shift in the distribution curve and increased peak intensity were observed at 0.45% SP dosage. This phenomenon corresponded to reduced ultimate tensile strain (0.95%). In the LC^3^-PE composite system, the enhanced dispersion of PE fibers was achieved through increased SP dosage, resulting in an elevation in ultimate tensile strain from 4.39% to 8.40%, with slight rightward shift in the crack-width distribution curve and a minimal 2 μm increase in average crack width (from 64.4 μm to 66.4 μm). These findings confirm the retention of superior crack-width control capability in LC^3^-PE specimens under high-strain conditions.

### 3.5. Carbon Emissions and Economic Analysis

As an important analytical tool in environmental management, life cycle assessments are widely recognized due to their systematic framework. The system boundary for ECC materials is defined as ‘cradle-to-gate’, encompassing processes from raw material extraction through transportation to the construction site. The carbon inventory mainly includes raw material acquisition and transport, kiln calcination, grinding processes, ECC mixing and transportation. Direct CO_2_ emissions comprise two primary components: the decomposition of raw materials at high temperature and fuel combustion from mining machinery and kiln operations. Indirect emissions cover the energy emission equivalent corresponding to the power consumption of production equipment and the emissions generated during the material transportation process.

The environmentally friendly advantages of LC^3^ cement are primarily attributed to the thermal activation of calcined clay at a lower temperature than that of Portland cement clinker in the calcination stage. Malacarne et al. [30] demonstrated that calcining 322.24 kg of lay at 800 °C in a rotary kiln using petroleum coke fuel yields 300 kg of calcined clay. This process requires 2.80 kg of petroleum coke, generating 9.6 kg of CO_2_ emissions from fuel combustion, indicating a rate of 32 kg of carbon emissions per ton of calcined clay produced. The indirect carbon emissions associated with calcined clay production include electricity-derived emissions from crushing, grinding and the calcination processes of the clay and transportation-related emissions. As established in Sánchez Berriel et al.’s research [6], the clay grinding process consumes 0.0249 kWh/kg of electrical energy. For the production of calcined clay, the energy consumption level of the rotary kiln is 0.079 KWh/kg, and the power demand for the grinding process is 0.05 kWh/kg. Material transportation utilizes 10 ton diesel trucks with a carbon emission factor of 0.162 kg CO_2_-eq/(t·km). As a result, the carbon emission levels of power equipment and transportation equipment are shown in Table 9.

As a coal combustion by-product, fly ash significantly transforms conventional solid waste management approaches in resource utilization. According to the 2025 Century Building Materials Monthly Report [31], the benchmark market price for fly ash in major Chinese cities is stabilized at 130 RMB/ton in early 2025. Regarding energy conversion efficiency, operational data from a Dalian thermal power enterprise demonstrate that generating 1 kWh of electricity requires 399 g of coal equivalent, simultaneously producing 109 g of fly ash. The allocation factor was calculated using Equation (4), yielding the production-stage carbon emission factor for fly ash presented in Table 10.

(4) $$C=\frac{{\left(\$\times m\right)}_{by\text{-}product}}{{\left(\$\times m\right)}_{main\text{-}product}+{\left(\$\times m\right)}_{by\text{-}product}}$$

where *C* is the distribution coefficient; $ × *m* is the product of product unit price and product quality in the production process.

The acquisition of carbon emission factors for other ECC constituents—Portland cement, limestone filler, silica sand and water—relies on well-established databases [32]. For superplasticizers and fibers, data availability is limited due to proprietary manufacturing processes or confidential material formulations. Direct carbon emissions for these materials were sourced from corresponding literature and specifications [33,34], which was determined as the average of the remaining values after removing the highest and lowest values.

Table 11 presents the life cycle environmental inventory for the LC^3^-ECC; the list analyzes the quantifying resource inputs, energy consumption and environmental emissions across the entire production using CO_2_-equivalent emissions as the unified metric. Additionally, each component’s manufacturing and transportation costs are listed. According to the local market price (Hangzhou city, China), the transportation price is 4 RMB/km/truck if the distance is more than 500 km and 5 RMB/km/truck if the distance is less than 500 km, assuming that the carrying capacity is 10 t/truck.

Based on the LC^3^-ECC mix design, the carbon emissions and costs of the hardened ECC composite per cubic meter were evaluated. Carbon emissions were calculated in accordance with the Chinese standard GB/T-51366-2019 [37]. The total carbon footprint comprises the sum of the production-stage and transportation-stage emissions, as expressed in Equation (5):

(5) $$C_{JC}=\frac{C_{SC}+C_{YS}}{V}$$

where *C_SC_* is the carbon emission per unit volume in the ECC production stage (kgCO_2_eq); *C_YS_* is the carbon emission per unit volume in the ECC transportation stage (kgCO_2_eq); *V* is the volume (m^3^).

Carbon emissions during the ECC production stage were quantified using Equation (6):

(6) $$C_{SC}=\sum_{i=1}^{n}M_{i}F_{i}$$

where *M_i_* is the i-th material consumption (t); *F_i_* is the i-th material carbon emission factor (kgCO_2_eq/unit material).

Carbon emissions during the concrete transportation stage were calculated using Equation (7):

(7) $$C_{YS}=\sum_{i}^{n}M_{i}D_{i}Y_{i}$$

where *D_i_* is the i-th transportation distance (km); *T_i_* is the carbon emission factor per unit distance of unit material transportation of the i-th material (kgCO_2_eq/(t·km)).

**Figure 16:**
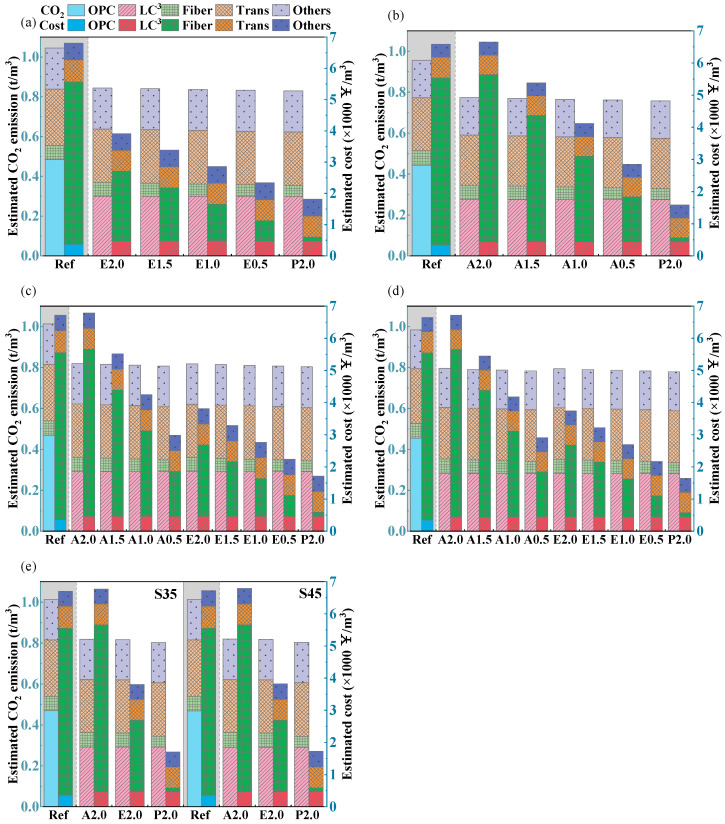
The unit carbon emissions and unit cost of LC^3^-ECC: (**a**) W22S60; (**b**) W30S20; (**c**) W25S40; (**d**) W27S30; (**e**) W25S35 and W25S45.

Figure 16 presents the carbon emissions and production costs per cubic meter of LC^3^-ECC. The reference group, comprising OPC-PVA composites with equivalent W/Bs and superplasticizer dosages, serves as the control for comparing conventional ECC and LC^3^-ECC formulations. Compared with the traditional ECC with OPC and PVA fiber (Ref. group), the carbon footprint of the LC^3^-ECC is reduced by 19.1–20.8%, due to the substitution of LC^3^ cement for Portland cement, leading to a significant reduction in the amount of Portland cement clinker [38,39]. Fibers contribute insignificantly to the total embodied carbon emissions. This is due to the small fraction of fibers in the ECC mixture.

Compared to carbon emissions, greater cost variations exist among different mix groups. This difference arises because fiber costs constitute a higher proportion of the total ECC production cost. In OPC-PVA mixtures, PVA fiber costs account for 76.3–79.0% of the total cost, while in LC^3^-PVA mixtures, this proportion ranges from 76.5% to 78.2%. When PE fibers were substituted with PP fibers, the fiber cost proportion decreased to 57.0–59.5%, resulting in a total cost reduction of approximately 43% compared to PVA fiber mixtures. Owing to the low-cost nature of PP fiber [40], further cost reductions are achieved when PVA or PE fibers are partially replaced by PP fibers. As the PP substitution rate increases, the total cost decreases nearly linearly. With full PP fiber replacement, the cost was reduced to approximately one-quarter of that of conventional ECC.

### 3.6. Multi-Dimensional Assessment of LC^3^-ECC

**Figure 17:**
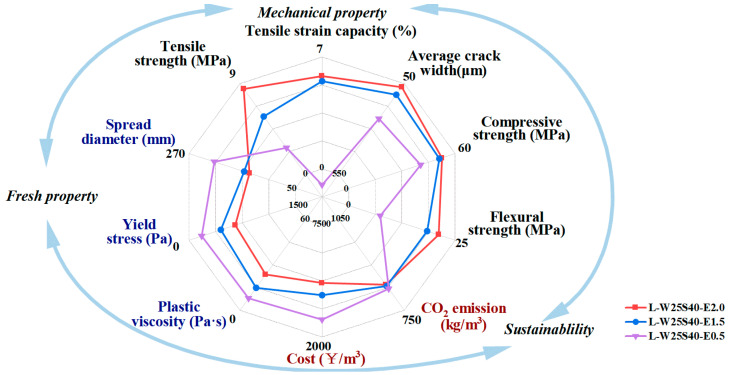
Comparison of working performance, mechanical properties and sustainability of LC^3^-PE/PP ECC.

Based on the three criteria, i.e., the rheology, mechanical properties and sustainability performance of ECC, an evaluation framework comprising eight indices was developed. Typical LC^3^-ECC mixes were selected to generate radar charts illustrating multi-criteria performance, as shown in Figure 17. In terms of rheology, it includes three indicators: fluidity, yield stress and plastic viscosity. In terms of mechanical properties, five indices, tensile strength, ultimate tensile strain, average crack width, compressive strength and flexural strength, were considered. Carbon emissions and production costs were used for characterizing sustainability. The polygon area enclosed by the radar chart axes and performance indicator vertices quantifies the comprehensive evaluation potential of an ECC system [14]. Larger polygon areas in mechanical, workability and sustainability domains generally enhance material advantages for structural applications. The optimal mix decision depends on the purpose and background of the specific construction design. For instance, for sustainable mix design purposes, carbon-reduction performance may be prioritized over maximizing mechanical properties when structural requirements permit. For infrastructure applications, a minimum tensile strain capacity of 2% satisfies most serviceability requirements. Regarding durability, maintaining crack widths below 50 μm preserves structural integrity under severe exposure conditions.

According to the results shown in Figure 17, CO_2_ emission was low for all the mixes because of the use of LC^3^ cement. The mix L-W25S40-E2.0 achieves the best mechanical performance with an ultimate tensile strain of 6.04% and average crack width of 65.7 μm, as well as the highest strengths. On the other hand, L-W25S40-E2.0 has the worst rheological performance. If a construction has a high requirement in rheological aspects, such as 3D printing materials, the mix L-W25S40-E0.5 is more proper, but the tensile performance and cracking resistance are greatly degraded. Additionally, the mix L-W25S40-E0.5 also owns the lowest cost. If the workability, mechanical properties and durability are all considered, the optimal mix is L-W25S40-E1.5. Its average crack width is 98.6 μm at 6% tensile strain, but the width is expected to be much smaller at normal working conditions such as 2% tensile strain. Moreover, previous studies [41] show that the self-healing of small cracks was observed in an LC^3^-based ECC exposed to NaCl solutions. L-W25S40-E1.5 achieves comparable mechanical strength to L-W25S40-E2.0, while L-W25S40-E1.5 has a lower cost and better workability.

**Figure 18:**
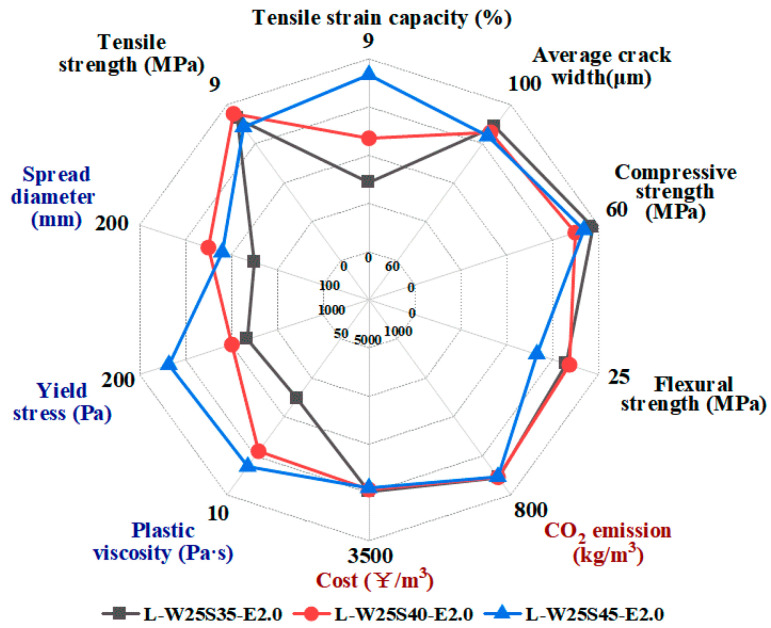
LC^3^-ECC performance radar chart under different SP contents.

The radar graph with multiple indicators can also be used to evaluate the effects of mix parameters on different properties. Figure 18 illustrates the influence of SP dosage on the properties of the LC^3^-ECC. The increase in SP dosage obviously improved the workability, with increased fluidity and reduced yield stress and plastic viscosity. The comparison also shows that increased SP content enhances the ultimate tensile strain of LC^3^-PE composites, probably due to improved fiber dispersion. When the SP dosage increases from 0.35% to 0.45%, the tensile and compressive strengths remain largely unaffected. On the other hand, the flexure strength was noticeably reduced as the SP dosage reached 0.45%. Additionally, SP dosage had insignificant effects on average crack width, CO_2_ emissions and cost.

## 4. Conclusions

In this paper, the mix design and performance optimization of the new low-carbon cementitious material system based on LC^3^-ECC were systematically studied. A comprehensive decision-making framework for optimal mix selection was constructed through an integrated material–mechanical–sustainability analysis framework. The main conclusions are as follows:

(1) The fluidity of OPC-ECC is better than that of LC^3^-ECC due to the high water demand of metakaolin in LC^3^ cement. PVA-ECC has higher fluidity and lower yield strength and plastic viscosity than that of PE-ECC because of different surface treatment. ECC containing PP fibers with the oil treatment has the highest fluidity and the lowest yield strength and plastic viscosity.
(2) The cracking patterns of ECC can be improved by increasing the water-binder ratio moderately, especially for the groups with PVA fiber. When the water-binder ratio is increased from 0.22 to 0.30, the number of cracks in LC^3^-PVA and OPC-PVA increases, and the crack width is smaller. The ultimate tensile strain is also increased, indicating an improved ductility.
(3) When PP fiber was blended into LC^3^-PE ECC, the fluidity increased with increasing PP replacement, while the yield stress and plastic viscosity decreased. In contrast, for the LC^3^-PVA system with PP, the yield stress and plastic viscosity showed no obvious trends as PP content increased. With increasing PP fiber replacement across all mixtures, flexural strength, compressive strength, tensile performance and crack-resistance were degraded. However, the LC^3^-PE specimen with 0.5% PP has a considerable ultimate tensile strength and even better ultimate elongation at a specific combination (W22S60 and W25S40).
(4) The carbon emission level of LC^3^-ECC is approximately 20% lower than that of conventional ECC (OPC-PVA), which is attributed to its lower cement clinker content. Different fiber types have slight effects on carbon emissions. The use of PP fiber resulted in significant cost reduction; the cost of LC^3^-PP is approximately 50% of that of LC^3^-PVA. Radar charts based on workability, mechanical properties and sustainability were established to assist researchers in selecting mixture proportions and identifying low-cost, environmentally friendly LC^3^-ECC formulations that achieve the desired workability and target strength.

For the mix design, the variables of this study are mainly concentrated in the water-binder ratio, the dosage of water reducing agent and the dosage of PP fiber. In the rheological test, excessive SP dosage will cause serious segregation and bleeding of the ECC paste. In future study, the combination of SP and thickening agent will be used to improve workability. Additionally, it is suggested to use digital image correlation (DIC) technology to detect crack development during tensile tests.

## Figures and Tables

**Table 1:** Chemical composition of cementitious materials (%).

Cementitious Materials	CaO	SiO_2_	Al_2_O_3_	Fe_2_O_3_	SO_3_	MgO	TiO_2_	CaCO_3_
P.I 42.5	64.16	20.96	4.64	3.04	2.07	3.15	0	0
MK	0.10	46.82	50.46	0.44	0	0.13	1.16	0
LS	0	0.02	0.01	0	0	0.11	0	98.50
FA	5.35	45.10	36.8	0.85	1.20	0	0	0

**Table 2:** Fiber property parameters.

Fiber	Length (mm)	Diameter (μm)	Density (g/cm^3^)	Tensile Strength (MPa)	Ultimate Elongation (%)	Elastic Modulus (GPa)
PVA	12	39	1.3	1833	6.7	41
PE	12	25	0.97	3100	3.5	122
PP	12	48	0.92	580	15	4.7

**Table 3:** Mix proportions of the ECC mortar matrix (kg/m^3^).

Number	PC	FA	MK	LS	Water	SP (%)	QS
O-W22S50	600	720	-	-	297	0.50	404
O-W22S60	600	720	-	-	297	0.60	404
O-W25S35	600	720	-	-	330	0.35	404
O-W25S40	600	720	-	-	330	0.40	404
O-W25S45	600	720	-	-	330	0.45	404
O-W27S30	600	720	-	-	363	0.30	404
O-W27S35	600	720	-	-	363	0.35	404
O-W27S40	600	720	-	-	363	0.40	404
O-W30S20	600	720	-	-	396	0.20	404
O-W30S30	600	720	-	-	396	0.30	404
O-W30S35	600	720	-	-	396	0.35	404
L-W22S50	330	720	180	90	297	0.50	404
L-W22S60	330	720	180	90	297	0.60	404
L-W25S35	330	720	180	90	330	0.35	404
L-W25S40	330	720	180	90	330	0.40	404
L-W25S45	330	720	180	90	330	0.45	404
L-W27S30	330	720	180	90	363	0.30	404
L-W27S35	330	720	180	90	363	0.35	404
L-W27S40	330	720	180	90	363	0.40	404
L-W30S20	330	720	180	90	396	0.20	404
L-W30S30	330	720	180	90	396	0.30	404
L-W30S35	330	720	180	90	396	0.35	404

**Table 4:** Tensile performance parameters of ECCs with different W/Bs.

Group	Initial Crack Strength (MPa)	Ultimate Tensile Strength (MPa)	Ultimate Tensile Strain (%)
O-W22S60-A	6.08 ± 1.06	7.87 ± 0.58	0.97 ± 0.22
L-W22S60-E	5.21 ± 0.46	8.17 ± 0.51	3.70 ± 0.85
L-W22S60-P	3.33 ± 0.43	3.33 ± 0.43	0.03 ± 0.01
O-W25S40-A	3.94 ± 0.14	6.71 ± 0.71	1.24 ± 0.42
L-W25S40-A	3.10 ± 0.73	7.28 ± 0.38	2.00 ± 0.43
L-W25S40-E	4.60 ± 0.5	8.59 ± 0.48	6.04 ± 0.42
L-W25S40-P	2.68 ± 0.35	2.68 ± 0.35	0.01 ± 0.00
O-W27S30-A	7.13 ± 1.66	8.40 ± 0.29	0.95 ± 0.19
L-W27S30-A	4.74 ± 0.55	7.18 ± 0.33	1.74 ± 0.72
L-W27S30-E	4.41 ± 0.40	7.89 ± 0.68	7.78 ± 1.12
L-W27S30-P	2.77 ± 0.17	2.77 ± 0.17	0.03 ± 0.07
O-W30S20-A	4.88 ± 1.14	7.09 ± 0.49	1.14 ± 0.22
L-W30S20-A	5.26 ± 1.07	6.29 ± 0.64	4.20 ± 0.44
L-W30S20-P	3.10 ± 0.63	3.10 ± 0.63	0.02 ± 0.00

**Table 5:** Tensile performance parameters of ECCs with different SP dosages.

Group	Initial Crack Strength (MPa)	Ultimate Tensile Strength (MPa)	Ultimate Tensile Strain (%)
O-W25S35-A	6.15 ± 0.52	8.17 ± 0.33	1.13 ± 0.31
O-W25S40-A	3.94 ± 0.14	6.71 ± 0.71	1.24 ± 0.42
O-W25S45-A	4.98 ± 0.96	5.68 ± 0.64	0.95 ± 0.30
L-W25S35-A	4.37 ± 0.56	6.76 ± 0.43	4.47 ± 0.44
L-W25S40-A	3.15 ± 0.73	7.28 ± 0.35	2.00 ± 0.43
L-W25S45-A	3.85 ± 0.33	4.88 ± 0.70	2.42 ± 0.11
L-W25S35-E	6.48 ± 0.57	8.36 ± 0.45	4.39 ± 0.64
L-W25S40-E	4.60 ± 0.50	8.59 ± 0.48	6.04 ± 0.42
L-W25S45-E	5.96 ± 1.20	7.94 ± 1.07	8.40 ± 0.42
L-W25S35-P	3.05 ± 0.38	3.05 ± 0.38	0.01 ± 0.00
L-W25S40-P	2.68 ± 0.35	2.68 ± 0.35	0.01 ± 0.00
L-W25S45-P	3.57 ± 0.21	3.57 ± 0.21	0.03 ± 0.01

**Table 6:** Tensile performance parameters of ECCs with different PP content.

Group	Initial Crack Strength (MPa)	Ultimate Tensile Strength (MPa)	Ultimate Tensile Strain (%)
L-W22S60-E	5.21 ± 0.46	8.17 ± 0.51	3.70 ± 0.85
L-W22S60-E1.5P	4.32 ± 0.59	7.14 ± 0.38	5.48 ± 1.35
L-W22S60-E1.0P	3.85 ± 0.52	4.55 ± 0.26	2.76 ± 0.88
L-W22S60-E0.5P	3.52 ± 0.19	3.52 ± 0.19	1.37 ± 0.46
L-W22S60-P	3.33 ± 0.43	3.33 ± 0.43	0.03 ± 0.01
L-W25S40-E	4.60 ± 0.5	8.59 ± 0.48	6.04 ± 0.42
L-W25S40-E1.5P	3.85 ± 0.23	6.39 ± 0.15	5.79 ± 1.86
L-W25S40-E1.0P	4.74 ± 0.64	4.74 ± 0.32	1.71 ± 0.76
L-W25S40-E0.5P	3.90 ± 0.63	3.90 ± 0.24	0.02 ± 0.01
L-W25S40-P	2.68 ± 0.35	2.68 ± 0.35	0.01 ± 0.00
L-W27S30-E	4.41 ± 0.40	7.89 ± 0.68	7.78 ± 1.12
L-W27S30-E1.5P	2.39 ± 0.24	5.68 ± 0.44	3.82 ± 0.87
L-W27S30-E1.0P	2.21 ± 0.72	4.37 ± 0.20	1.62 ± 0.55
L-W27S30-E0.5P	2.68 ± 0.12	2.68 ± 0.12	0.88 ± 0.05
L-W27S30-P	2.77 ± 0.17	2.77 ± 0.17	0.03 ± 0.07
L-W25S40-A	3.10 ± 0.73	7.28 ± 0.38	2.00 ± 0.43
L-W25S40-A1.5P	2.91 ± 0.80	5.63 ± 0.92	0.54 ± 0.12
L-W25S40-A1.0P	2.96 ± 0.22	3.10 ± 0.12	0.24 ± 0.04
L-W25S40-A0.5P	1.64 ± 0.55	1.83 ± 0.35	0.19 ± 0.02
L-W25S40-P	2.68 ± 0.35	2.68 ± 0.35	0.01 ± 0.00
L-W27S30-A	4.74 ± 0.55	7.18 ± 0.33	1.74 ± 0.72
L-W27S30-A1.5P	4.46 ± 0.15	4.88 ± 0.50	0.32 ± 0.06
L-W27S30-A1.0P	3.85 ± 0.13	4.46 ± 0.54	0.36 ± 0.05
L-W27S30-A0.5P	2.30 ± 0.43	2.30 ± 0.38	0.37 ± 0.03
L-W27S30-P	2.77 ± 0.17	2.77 ± 0.17	0.03 ± 0.07
L-W30S20-A	5.26 ± 1.07	6.29 ± 0.64	4.20 ± 0.44
L-W30S20-A1.5P	3.33 ± 0.26	5.49 ± 0.12	1.40 ± 0.21
L-W30S20-A1.0P	2.54 ± 1.35	4.08 ± 0.35	1.63 ± 0.59
L-W30S20-A0.5P	3.24 ± 0.27	3.24 ± 0.27	0.03 ± 0.01
L-W30S20-P	3.10 ± 0.63	3.10 ± 0.63	0.02 ± 0.00

**Table 7:** The distribution of tensile crack widths.

Group		Average Crack Width μm	Fracture Number									
			0–15 μm	15–30 μm	30–45 μm	45–60 μm	60–75 μm	75–90 μm	90–105 μm	105–120 μm	120–200 μm	>200 μm
W22 S60	L-E	46.5	3	11	11	4	2	6	1	1	1	0
	L-E1.5P	75.7	2	3	7	5	2	3	2	0	7	1
	L-E1.0P	158.1	0	0	0	0	2	0	2	1	0	3
	L-E0.5P	159.3	0	0	0	0	0	0	0	0	3	0
W25 S35	O-A	62.8	0	1	5	0	0	0	1	0	2	0
	L-A	44.4	3	19	13	4	4	1	0	1	0	1
	L-E	64.4	0	9	12	6	5	3	2	1	1	1
W25 S40	L-E	65.7	0	10	12	7	7	5	1	3	4	2
	L-E1.5P	98.6	0	1	5	3	4	2	2	3	8	2
	L-E1.0P	216.3	0	0	0	0	0	0	0	0	2	3
	L-A	61.7	1	8	3	2	1	3	1	0	0	1
	O-A	79.9	0	2	1	1	1	1	1	0	1	1
W25 S45	O-A	44.6	4	2	0	3	0	0	0	0	1	0
	L-A	54.8	3	16	4	0	0	0	0	0	0	1
	L-E	66.4	1	14	14	14	9	7	5	5	2	2
W27 S30	L-E	56.9	2	24	12	7	7	7	8	1	5	1
	L-E1.5P	120.0	0	0	1	1	1	1	3	3	5	1
	L-E1.0P	212.2	0	0	0	0	0	1	0	0	1	2
	L-E0.5P	176.9	0	0	0	0	0	0	0	0	2	1
	L-A	60.8	0	4	2	4	1	1	1	0	0	1
W30 S20	L-A	36.1	2	26	5	5	1	1	1	0	2	0
	L-A1.5P	124.6	0	0	0	2	1	0	1	0	2	1
	L-A1.0P	123.1	0	0	0	0	2	1	1	0	1	1
	O-A	28.2	1	15	5	1	0	1	0	0	0	0

**Table 8:** Fitted Weibull distribution parameters for crack widths.

Group		Scale Parameter *λ*	Lower Confidence Limit *λ*_l_	Upper Confidence Limit *λ*_u_	Shape Parameter *k*	Lower Confidence Limit *k*_l_	Upper Confidence Limit *k*_u_
W22 S60	L-E	52.6	42.7	62.0	1.75	1.53	2.19
	L-E1.5P	84.4	63.6	104.3	1.53	1.28	1.93
	L-E1.0P	179.2	104.9	260.1	1.77	1.47	5.39
	L-E0.5P	169.0	143.1	176.5	7.88	1.89	25.46
W25 S35	O-A	70.7	38.9	104.1	1.62	1.34	4.58
	L-A	46.9	34.9	62.2	1.11	0.89	2.39
	L-E	68.0	40.6	83.5	1.12	0.99	1.49
W25 S40	L-E	73.7	61.2	86.6	1.53	1.33	2.08
	L-E1.5P	111.7	90.9	135.3	1.93	1.65	2.62
	L-E1.0P	239.4	183.3	288.0	3.97	2.77	14.20
	L-A	63.0	39.3	98.0	1.04	0.84	2.25
	O-A	89.3	53.9	138.9	1.49	1.17	2.98
W25 S45	O-A	44.8	21.2	82.4	1.01	0.84	2.10
	L-A	55.1	24.0	64.3	1.01	0.70	4.49
	L-E	73.01	61.8	87.3	1.31	1.18	1.66
W27 S30	L-E	60.3	53.4	75.0	1.22	1.10	1.72
	L-E1.5P	135.6	107.4	165.4	2.40	1.87	4.41
	L-E1.0P	239.4	140.7	318.1	2.63	1.74	13.48
	L-E0.5P	189.9	164.0	197	6.31	2.84	18.35
	L-A	66.2	43.6	100.2	1.25	1.01	3.19
W30 S20	L-A	40.1	31.4	50.1	1.38	1.18	2.02
	L-A1.5P	140.1	80.8	219.0	1.56	1.23	4.37
	L-A1.0P	139.9	83.9	218.2	1.81	1.56	6.50
	O-A	32.0	25.8	38.8	2.11	1.75	4.75

**Table 9:** Indirect carbon emission coefficient of calcined clay in the production and transportation processes.

	Electrical Equipment (kgCO_2_/kg)			Transportation Systems (kgCO_2_/t/km)
	Crushing	Calcination	Grinding	
Carbon emission factor	0.0288	0.0914	0.0579	0.1620

**Table 10:** Product distribution coefficient of a thermal power plant.

Product	Output	Market Price	Distribution Coefficient According to Economic Value (%)	Carbon Emission Factor (kgCO_2_eq/t)
Electric power	1 kwh	0.5 RMB/kwh	97.24	-
Fly ash	0.109 kg	130 RMB/ton	2.76	272.78

**Table 11:** Environmental impact inventory and component cost of the LC^3^-ECC [10,20,22,25,32,33,34,35,36].

Component	Carbon Emission (kgCO_2_/kg)			Cost (RMB/kg)			Transportation Distance (km)
	Production	Transportation	Total	Production	Transportation	Total	
PI 42.5	0.841	0.211	1.051	0.636	0.520	1.156	1300
MK	0.188	0.149	0.337	1.200	0.368	1.568	920
LS	0.018	0.149	0.167	0.798	0.368	1.166	920
FA	0.273	0.149	0.422	0.130	0.368	0.498	920
SS	0.023	0.149	0.172	0.798	0.368	1.166	920
SP	1.196	0.300	1.496	18.830	0.740	19.570	1850
W	0.001	0.000	0.001	0.004	0.000	0.004	0
PVA	2.724	0.032	2.756	200.000	0.080	200.080	200
PE	3.560	0.049	3.609	115.000	0.120	115.120	300
PP	2.900	0.130	3.030	7.000	0.320	7.320	800

Note: data from the literature retain the average value calculated after removing the highest and lowest values.

## Data Availability

The original contributions presented in this study are included in the article. Further inquiries can be directed to the corresponding author.
